# Chronic urticaria: profile from a reference center^☆^

**DOI:** 10.1016/j.abd.2021.01.006

**Published:** 2022-06-12

**Authors:** Ana Carolina Miranda Carvalho Ferreira Fernandes de Souza, Sérgio D. Dortas Junior, Guilherme Gomes Azizi, Alfeu Tavares França, Omar Lupi, Solange O.R. Valle

**Affiliations:** Hospital Universitário Clementino Fraga Filho, Rio de Janeiro, RJ, Brazil

Dear Editor,

Urticaria is a common condition, determined by the activation of mast cells, presenting clinically as wheals, angioedema, or both.^1^ It is a heterogeneous disease which is easily recognized by patients and physicians. However, it is highly complex when considering the etiology and therapies. It was agreed to define urticaria, in terms of its duration, in two forms: acute (AU) and chronic (CU). Urticaria is defined as chronic when it persists for six weeks or longer.^1^ Chronic urticaria comprises chronic spontaneous urticaria (CSU) and chronic inducible urticaria (CIndU), which include physical and non-physical urticaria.^2^

The prevalence of physical urticaria (PU) in adults varies from 20% to 30% among urticaria cases and 6.2% to 25.5% in children. It is estimated that PU is present in up to 5% of the general population. In 10% to 50% of patients with CU, at least one type of PU is identified (most often symptomatic dermographism and delayed pressure urticaria).^3^

It is important to recognize that associations of CIndU and CSU are often observed, whereas a patient may simultaneously have two or more forms of CIndU. Patients with CSU with a CIndU component have a worse prognosis, with longer disease duration.^4^, ^5^ For instance, in a study by Kozel et al., the one-year remission rate in patients with an association of CSU and CIndU was 21%, compared with 47% in patients with CSU alone.^6^

CIndUs can be diagnosed through clinical history, physical examination, and lesion reproduction using challenge tests.^2^

A retrospective study was carried out by analyzing the medical records of 179 patients with a history of CU, followed at a Urticaria Center of Reference and Excellence (GA^2^LEN UCARE),^7^ from 2015 to 2019. The main goals of the GA²LEN UCAREs are to provide excellent care in urticaria management, increase urticaria knowledge through research and education, and promote urticaria awareness. To become a certified GA²LEN UCARE, urticaria centers must meet 32 ​​requirements that are assessed during an audit visit.^7^

The epidemiological profile of these patients was analyzed, and the following parameters were assessed: sex, age, disease duration, presence of CSU and/or CIndU and CIndU subtype. CIndU subtypes were confirmed through validated challenge tests.^2^

Of the analyzed medical records, 153 (86%) were from females and 26 (14%) from male patients. The mean age was 46.3 years (6–81 years), and disease duration averaged 10.2 years. Ninety-seven (54%) patients had an association between CSU and CIndU, 63 (35%) had only CSU, and 19 (11%) had only CIndU (Fig. 1). Among the patients who had only CIndU, whether only one form or several forms of CIndU per patient, the subtypes were: 12 (50%) with dermographism, four (18%) with delayed pressure urticaria (DPU), one (4%) with cold urticaria, two (8%) with heat urticaria, two (8%) with cholinergic urticaria, one (4%) with vibratory urticaria, one (4%) with solar urticaria and one (4%) with aquagenic urticaria. Among the patients with CIndU associated with CSU, whether only one form or several forms of CIndU per patient, the subtypes were: 81 (86%) with dermographism, 27 (29%) with DPU, four (4.3%) with cold urticaria, eight (8.6%) with heat urticaria, one (1.1%) with solar urticaria and nine (9.6%) with cholinergic urticaria (Fig. 2).

**Figure 1:**
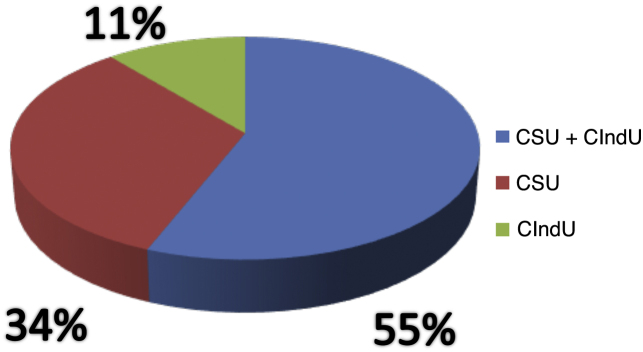
Profile of patients with urticaria.

**Figure 2:**
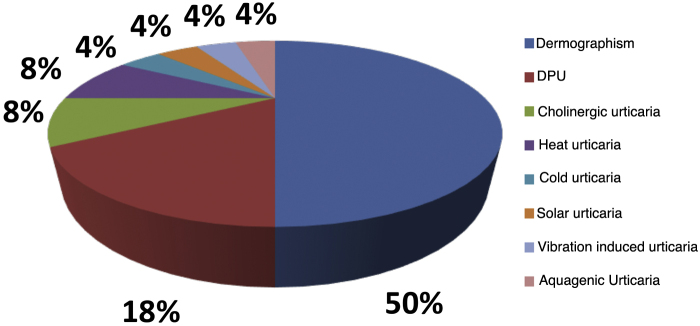
Profile of patients with inducible chronic urticaria.

The present data corroborate the findings of other epidemiological studies, with a higher prevalence of CU in females, age group (20‒30 years), an association between CSU and CIndU, in addition to the most frequent type of CIndU (dermographism). On the other hand, different from the findings of European studies, the authors found a longer disease duration in the studied group (10.2 years × 3-5 years).^8^, ^9^ This difference seems to be related to the high prevalence of CIndU in the assessed sample.

Finally, the identification and management of CIndUs in patients with CU are extremely relevant since they are associated with a worse prognosis and longer disease duration, which negatively affects patient quality of life.

## Financial support

None declared.

## Authors' contributions

Ana Carolina Miranda Carvalho Ferreira Fernandes de Souza: Approval of the final version of the manuscript; design and planning of the study; collection, analysis, and interpretation of data; intellectual participation in the propaedeutic and/or therapeutic conduct of the studied cases.

Sérgio D. Dortas Junior: Approval of the final version of the manuscript; design and planning of the study; collection, analysis, and interpretation of data; intellectual participation in the propaedeutic and/or therapeutic conduct of the studied cases; critical review of the literature; critical review of the manuscript; drafting and editing of the manuscript.

Guilherme Gomes Azizi: Approval of the final version of the manuscript; data interpretation; intellectual participation in the propaedeutic and/or therapeutic conduct of the studied cases; critical review of the literature; critical review of the manuscript; statistical analysis.

Alfeu Tavares França: Approval of the final version of the manuscript; intellectual participation in the propaedeutic and/or therapeutic conduct of the studied cases; critical review of the literature; critical review of the manuscript; effective participation in research orientation.

Omar Lupi: Approval of the final version of the manuscript, critical review of the literature, critical review of the manuscript, effective participation in research orientation.

Solange O R Valle: Approval of the final version of the manuscript; intellectual participation in the propaedeutic and/or therapeutic conduct of the studied cases; critical review of the literature; critical review of the manuscript; effective participation in research orientation.

## Conflicts of interest

None declared.

## References

[1] Zuberbier T., Aberer W., Asero R., Latiff A.H.A., Baker D., Ballmer-Weber B. (2018). The EAACI/GA^2^LEN/EDF/WAO guideline for the definition, classification, diagnosis and management of urticaria. Allergy.

[2] Magerl M., Altrichter S., Borzova E., Giménez-Arnau A., Grattan C.E.H., Lawlor F. (2016). The definition, diagnostic testing, and management of chronic inducible urticarias – TheEAACI/GA(2) LEN/EDF/UNEV consensus recommendations 2016 update and revision. Allergy.

[3] Weller K., Altrichter S., Ardelean E., Krause K., Magerl M., Siebenhaar F. (2010). Chronic urticaria. Prevalence, course, prognostic factors andimpact. Hautarzt.

[4] Sánchez-Borges M., González-Aveledo L., Caballero-Fonseca F., Capriles-Hulett A. (2017). Review of Physical Urticarias and Testing Methods. Curr Allergy Asthma Rep.

[5] Kozel M.M., Mekkes J.R., Bossuyt P.M., Bos J.D. (2001). Natural course of physical and chronic urticaria and angioedema in 220 patients. J Am Acad Dermatol.

[6] Kaplan A.P., Gray L., Shaff R.E., Horakova Z., Beaven M.A. (1975). In vivo studies of mediator release in cold urticaria and cholinergic urticaria. J Allergy Clin Immunol.

[7] Maurer M., Metz M., Bindslev-Jensen C., Bousquet J., Canonica G.W., Church M.K. (2016). Definition, aims, and implementation of GA(2)LEN urticaria centers of reference and excellence. Allergy.

[8] Gaig P., Olona M., Lejarazu D.M., Caballero M.T., Domínguez F.J., Echechipia S. (2004). Epidemiologyof urticaria in Spain. J Investig AllergolClin Immunol.

[9] Lapi F., Cassano N., Pegoraro V., Cataldo N., Heiman F., Cricelli I. (2016). Epidemiology of chronic spontaneous urticaria: results from a nationwide, population-based study in Italy. Br J Dermatol.

